# Iron Oxide and Iron Sulfide Films Prepared for Dye-Sensitized Solar Cells

**DOI:** 10.3390/ma13081797

**Published:** 2020-04-11

**Authors:** Kostyantyn Tuharin, Zdeněk Turek, Michal Zanáška, Pavel Kudrna, Milan Tichý

**Affiliations:** 1 Faculty of Mathematics and Physics, Charles University, 121 16 Prague 2, Czech Republic; tuharink@gmail.com (K.T.); zdenek.turek11@gmail.com (Z.T.); zanaska@fzu.cz (M.Z.); Milan.Tichy@mff.cuni.cz (M.T.); 2 Institute of Physics of the Czech Academy of Sciences, 182 21 Prague 8, Czech Republic

**Keywords:** hollow cathode plasma jet, iron oxide, iron sulfide, absorption spectroscopy, dye-sensitized solar cell

## Abstract

In this paper, the prospects of iron oxide films and their sulfidation for dye-sensitized solar cells (DSSC) are reviewed. Iron oxide thin films were prepared by hollow cathode plasma jet (HCPJ) sputtering, with an admixture of oxygen in the argon working gas and with an iron nozzle as the sputtering target. The discharge was powered by a constant current source in continuous mode and by a constant voltage source in pulsed mode. Plasma composition was measured by an energy-resolved mass spectrometer. Moreover, secondary electron microscopy (SEM), energy-dispersive X-ray spectroscopy (EDX), absorption and Raman spectra of the films are presented. Strong correlation between the color of the iron oxide film and its phase composition was revealed. Iron oxide films were sulfided at 350 °C. A relatively clean pyrite phase was obtained from the magnetite, while the marcasite with admixture of the pyrite phase was obtained from the hematite. Low influence of sulfidation on the films’ microstructure was demonstrated.

## 1. Introduction

One of the biggest problems facing mankind is the search for substitutes for slowly but inevitably disappearing fossil fuels. At the same time, it is more necessary than ever to find ways to solve the negative consequences of the current energy system with regard to climate, environment and health. Of course, the quality of human life depends to a large extent on the availability of clean energy sources. One such source is solar power by means of solar batteries, especially dye-sensitized solar cells (DSSC).

**Figure 1:**
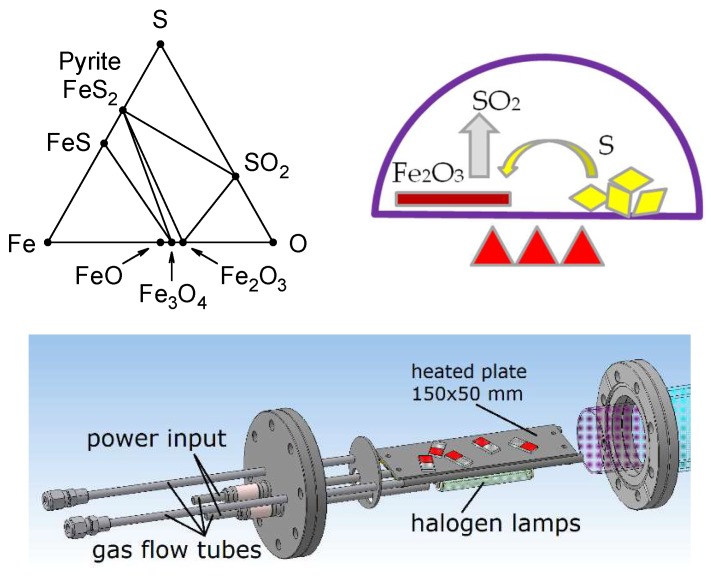
Fe-O-S phase diagram (**top left**), schematic of iron oxide sulfidation at a temperature of about 350 °C and atmospheric pressure (**top right**); and 3D model of the vacuum mini furnace for the iron oxide sulfidation of samples used in this article (**bottom**).

Iron oxide can serve as a convenient precursor for iron sulfide (FeS_2_), also known as pyrite, which is becoming a popular object of investigation nowadays. Pyrite is nontoxic, abundant and can be used in photovoltaic cells to enhance the photo-response of the front transparent electrode, or as an efficient low-cost counter electrode material potentially able to replace precious platinum [1,2]. The band gap energy of FeS_2_ ~1.0 eV is close to the ideal band gap range of 1.1–1.5 eV for a single-junction photovoltaic device [3]. Unfortunately, the traditional pyrite synthetic process could lead to unwanted phases during pyrite formation, such as FeS or other intermediate phases [1,4]. These conductive metallic phases destroy the semi-conductive properties of FeS_2_, reducing the quality of the resultant material. The conversion of the FeS phase to FeS_2_ is possible by chemical methods [5], but attention must be paid to avoid FeS residuals. The Fe-O-S phase diagram (see Figure 1, top left [6]) shows that pyrite synthesis in the absence of oxygen crosses the FeS phase. However, if sulfur is added to the oxides Fe_3_O_4_ or Fe_2_O_3_, one does not enter the FeS phase field. The substitution of oxygen with sulfur can be performed relatively easily in a furnace, with sulfur vapors (see Figure 1). The convenient temperature at atmospheric pressure is above 350 °C [7]. A similar method was used, e.g., in [8]. The substitution reaction can be described by the equations (where (s) and (g) stand for “solid” and “gas”, respectively):

2 Fe_2_O_3_ (s) + 11 S → 4 FeS_2_ (s) + 3 SO_2_ (g) (1)

Fe_3_O_4_ (s) + 8 S → 3 FeS_2_ (s) + 2 SO_2_ (g) (2)

Iron oxide thin films can be prepared by plasma deposition in magnetrons, electric arc sprayers and other methods [9]. We utilized a hollow cathode plasma jet with an argon working gas and an oxygen admixture. The iron is sputtered from an iron nozzle, which serves as the sputtering target. The anomalous glow discharge inside the hollow cathode is able to reach a much higher degree of ionization compared to a normal glow discharge. The so-called hollow cathode effect arises from the difference between exponential electron multiplication in the cathode sheath and linear multiplication in the plasma [10]. The consequence of the higher plasma density is a higher deposition rate. The important advantage of the hollow cathode system is the possible separate injection of the oxygen out of the nozzle. This way the target poisoning is eliminated, the discharge works in metallic mode and the deposition rate remains high. This also improves the discharge stability, especially in the pulsed mode.

## 2. Experimental Setup

**Figure 2:**
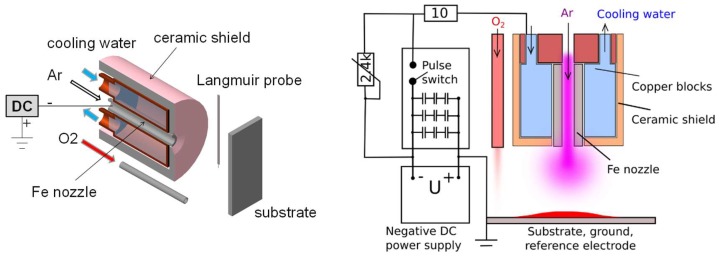
Cross-section of the hollow cathode sputtering system with DC power supply (**left**) and a schematic of the same hollow cathode with electric circuit for pulsed DC operation mode (**right**). Both systems are depicted without the vacuum chamber.

The hollow cathode plasma jet (HCPJ) is installed in the high vacuum chamber. The pure Fe (99.96%) nozzle is surrounded by copper blocks cooled by a water flow. While the argon is introduced into the system via the hollow cathode, the oxygen inflow tube is positioned a few centimeters away from the cathode (see Figure 2). The distance between the nozzle exit and the substrate is variable, 4–7 cm. The substrates were glass, silicon wafer and fluorine doped tin oxide (FTO) glass. The FTO film on the glass has the sheet resistance of 7 ohm/sq. This FTO film functions as a transparent electrode and provides the parallel conductivity to the contact in the assembled solar cell.

The outer surface of the hollow cathode system is insulated by a cylindrical ceramic shield. The discharge is powered by a constant current source in continuous mode or by a constant voltage source in pulsed mode. The apparatus was described in detail in previous studies [11]. A resistor in parallel with the pulse switch provides low continuous DC power, which is used as pre-ionization and achieves a reliable ignition of the pulsed discharge [12].

An oil-free vacuum system is pumped down to ultimate pressure on the order of 10^−4^ Pa by a combination of a turbomolecular pump and a piston pump. Prior to plasma ignition, the flow rate of the working gases is adjusted using mass flow controllers. A typical argon flow rate is between 100 and 300 sccm. The required pressure in the chamber is set by throttling the pumping speed by the gate valve between the chamber and the turbomolecular pump. During the deposition, the pressure inside the reaction chamber was in the range from 1 to 50 Pa. In order to easily ignite the discharge, the chamber pressure was temporarily increased to about 100 Pa, especially at pulsed operation [9].

## 3. Results and Discussion

### 3.1. Mass Spectrometric Diagnostics

To check the plasma composition, a Hiden EQP 500 mass spectrometer (Hiden Analytical, Warrington, UK) equipped with an energy analyzer was used. Its sampling orifice was positioned at the nozzle axis, at the location of the substrate holder, i.e., 6–7 cm from the nozzle, and was grounded. We compared the plasma parameters in continuous DC and pulsed DC modes when setting the ion energy analyzer to 0.5 eV. Table 1 shows the amplitudes of the strongest ion signal peaks identified in the mass spectrum with amplitudes above 1%. The indicated values are relative to that of the most abundant Ar^+^ ion. The signals for the isotopes ^36^Ar, ^54^Fe and ^57^Fe of argon and iron correspond to their relative natural abundances of 0.3%, 5.8% and 2.1%, respectively, and are not shown in the table. The mass 1 amu was not measured, because of its proximity to mass 0, which allows all ions to pass. The doubly charged Ar^++^ ion created from Ar^+^ is always present in the pure argon, with an amplitude above 11%. The ArH^+^ ions are formed from Ar^+^ and water impurities. This destruction reaction of Ar^+^ competes with the natural decay via diffusion. Therefore, the increase of the ArH^+^ percentage at a pressure of 20 Pa can be qualitatively explained by the overabundance of the ArH^+^ formation over about four times slower diffusion at 20 Pa compared to that at 5 Pa. Amplitudes of H_3_^+^ ions appear in an approximate proportion to ArH^+^, which corresponds to their probable creation from ArH^+^ and hydrogen.

The addition of oxygen changes the spectrum dramatically. The relative percentage of Ar^++^ decreases down to about 3%, and O_2_^+^ ions appear with relative amplitude of 55%. O_2_H^+^ ions seem to be formed from O_2_^+^ in the same way as ArH^+^ from Ar^+^, i.e., from the reaction of O_2_^+^ with the remaining traces of water. Fe^+^ ions are present with amplitudes of about 1.5% with respect to Ar^+^, at a pressure of 5 Pa. At 20 Pa, the Fe^+^ amplitude is much higher, around 12%. This corresponds to a higher Fe sputtering rate caused by an increase of the plasma density during the whole pulsed discharge period at higher pressure, as measured in [11].

**Figure 3:**
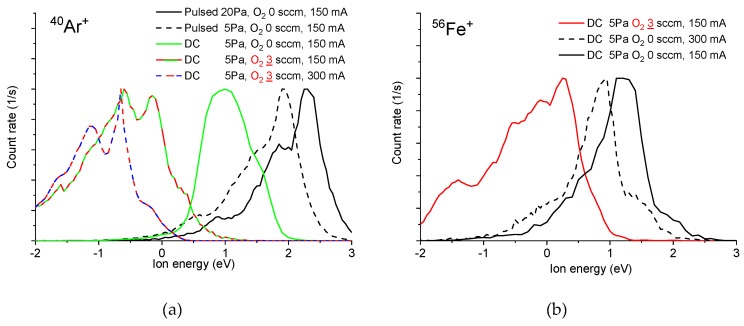

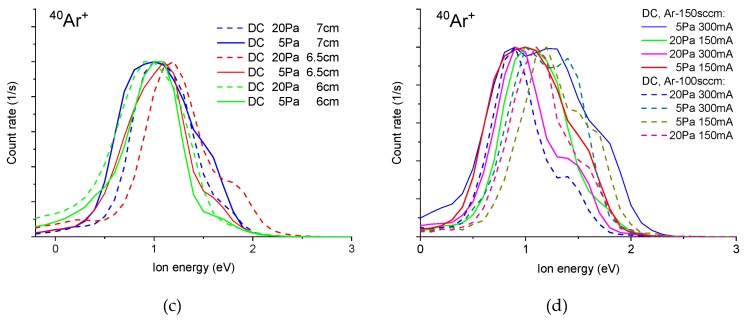
Energetic spectra for argon in DC and pulsed DC modes (**a**) and iron in DC mode (**b**). Energetic spectra for argon in DC mode for different pressures and distances from cathode (**c**) and argon flow rates (**d**). Default parameters: distance from cathode 7 cm, Ar flow 150 sccm, O_2_ flow 0 sccm, discharge current 150 mA and pulsed mode duty cycle 10% at frequency 1 kHz.

Energetic spectra of Ar^+^ and Fe^+^ positive ions measured by the mass spectrometer with the energetic analyzer are shown in Figure 3. It is seen that all measured energies are below 3 eV. Since the ion mean free path is in the range of 1.6 to 0.4 mm, at a pressure range of 0.5 to 20 Pa, the ions undergo many collisions before entering the mass spectrometer. For this reason, their speed is thermalized and the measured energies reflect mainly the value of the plasma potential. In the DC mode with pure argon without oxygen, the changes of the discharge current, argon flow rate, pressure and distance from the cathode influence the ion energy only slightly. The shifts are less than 0.5 eV (see bottom row of Figure 3). The highest energies with peak value up to 2.3 eV were measured for Ar^+^ ions in pulsed mode (see the top left panel of Figure 3). This is connected with the temporal dependence of the plasma potential, which nevertheless stays positive during the whole period of the pulsed discharge.

The addition of 3 sccm of oxygen to the working gas inside the reaction chamber leads to a significant decrease in the measured ion energies in DC mode (see top row of Figure 3 with Ar^+^ and Fe^+^ ions). The energy of the lowest measured energetic peak of Ar^+^ is about −1 eV. This apparently negative ion energy can be explained by the shift of the plasma potential to negative values after oxygen addition. This shift was measured in [13]. Although the sampling orifice of the spectrometer is grounded, it is covered by a nonconducting layer of iron oxides which are negatively charged. The negative charge attracts positive ions toward the sampling orifice. Since the mass spectrometer references the ion energy to ground, the positive ions coming from the negative plasma potential appear to have negative energies.

### 3.2. Thin Film Properties

**Figure 4:**
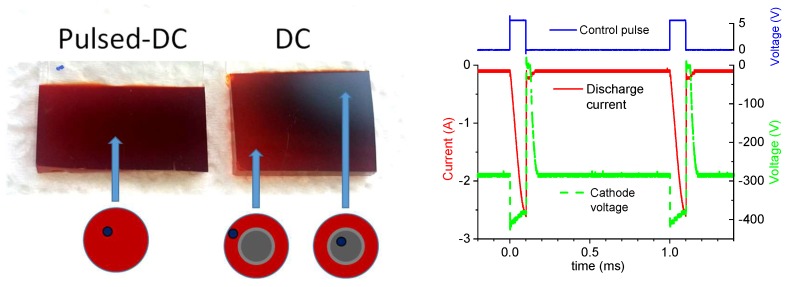
Photos of homogenous (pulsed-DC) and non-homogeneous (DC) iron oxide films on FTO glass substrate (**left**). The centers of schematic red circles correspond to the axis of the nozzle. Dark dots indicate the positions shown by arrows on the substrate. The discharge voltage and current waveforms during a typical pulsed DC operation with duty cycle of 10% and repetition frequency of 1 kHz are shown (**right**).

Iron oxide films created in the continuous DC mode show different properties in the central part corresponding to the hollow cathode axis and in the off-center part contrary to the pulsed DC mode that produced a homogeneous film (see Figure 4). There are sixteen known iron oxides and oxyhydroxides, the best known of which is rust, a form of iron (III) oxide [14]. After the deposition of iron with various oxygen concentrations and plasma parameters, we obtained films with dominating magnetite (Fe_3_O_4_) and hematite (Fe_2_O_3_) phases, as measured by Raman spectroscopy (see below). In the pulsed DC case, the hematite dominated the whole substrate surface; in the continuous DC case, magnetite dominated in the central part of the substrate; and in the peripheral parts of the substrate, hematite dominated. This is schematically indicated by color-coding below the photos in Figure 4: hematite and magnetite are denoted by red and black, respectively. The corresponding positions of the color-coded regions on the deposited films are indicated by blue arrows. The real color of the obtained samples was similar to that of natural minerals, i.e., it varied between black, yellow, red and brown.

After the deposition of iron oxide, the sulfidation of the samples was performed in the furnace shown in Figure 1. The iron oxide samples were inserted into the furnace chamber, with a small amount of sulfur powder. The chamber was then evacuated and filled with argon, to a pressure of 0.5 bar. Next, the heating using halogen bulbs was switched on, which gradually increased the temperature inside the chamber up to 350 °C over a period of approximately 60 min, completing the process of sulfidation. After switching off the heating, the temperature eased down to 230 °C in about 20 min.

**Figure 5:**
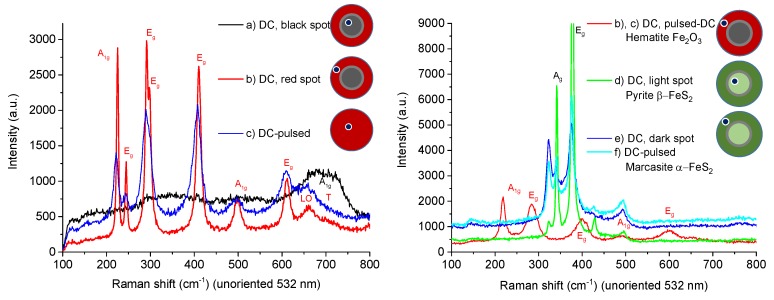
Raman spectra of iron oxide films before sulfidation, (a), (b) and (c) (**left**). Samples after sulfidation, (d), (e) and (f) (**right**). Amplitudes of measured spectra are scaled for better visibility.

The recorded Raman spectra are depicted in Figure 5, and the dominating phases identified in the spectra are listed in Table 2. The positions of the individual phases on the substrate with respect to the nozzle axis are indicated by dots on red circles for iron oxides and dots on green circles for iron sulfides. The pyrite phase (d) was obtained from magnetite (a), while the mix of marcasite and pyrite phase, (e) and (f), was obtained from hematite (b) and (c). Both sulfide phases are attractive for studying photovoltaic applications [15]. Other phases of iron sulfide were not identified. The Raman spectra after sulfidation in the right panel of Figure 5 show no traces of iron oxide phases, which indicates almost complete sulfidation up to the depth of the Raman diagnostics. Despite this, the EDX diagnostics showed a residual amount of oxygen listed in Table 3. This oxygen (22.4 atomic % for Marcasite and 20.5 at.% for Pyrite) cannot be completely attributed to SnO_2_ in FTO, because the amount of detected Sn is only 5.8 at.% for Marcasite and is almost negligible for Pyrite. Since the silicon was not detected, the electron penetration depth seems to be comparable to the film thickness. That means that the oxygen detected by EDX is attributed to the bulk of sulfide films below the detection depth of the Raman diagnostic and only partly attributed to SnO_2_ in the case of thinner Marcasite film.

**Figure 6:**
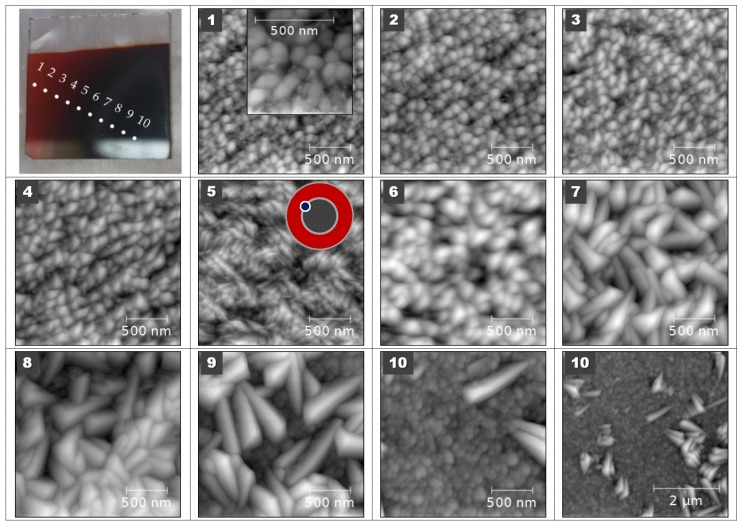
AFM images of iron oxide nanocrystals measured at different positions 1 to 10 marked on a thin film photograph (top left). Image at position 10 shown with two different magnifications. Film was deposited on a glass substrate in DC mode at discharge current 300 mA, voltage 296 V, pressure 4.6 Pa, distance from the nozzle 4 cm, argon flow rate 170 sccm and oxygen flow rate 1 sccm.

The morphology of both the iron oxide and iron sulfide films was studied by AFM and SEM. The obtained AFM images are shown in Figure 6. This figure shows 10 AFM images of approximate size 2 × 2 µm arranged sequentially at distances approximately 1.4 mm from the periphery towards the center of the substrate (10 corresponds to the system axis). It is seen that a wide range of possible nanostructures could be obtained using our experimental setup at DC regime. In AFM images 1–4 we see an increase of particle size and a slight change in their shape from round to triangular. AFM image number 5 corresponds to the interface between the black central part and the surrounding red peripheral ring and shows a different structure. The difference in interface structure at the boundary between the central and the outer part of the substrate and changing from sample to sample was observed at all samples with magnetite and hematite phases. The black central part, images 6–10, contains even bigger triangular nanocrystals growing on top of a dense film of smaller particles.

We observed that deposition in the DC mode is much more sensitive to experimental conditions than the pulsed-DC. Even a slight change of deposition parameters could lead to dramatic change of color, roughness, or phase structure of films and linear size of nanocrystals. The pulsed DC sputtering leads to homogenous hematite films at a wider range of parameters.

**Figure 7:**
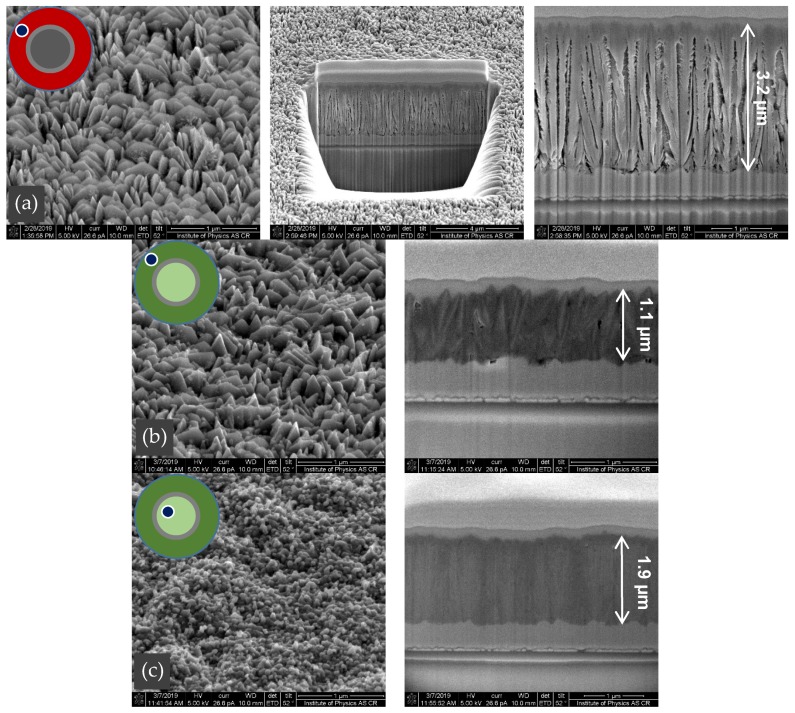
SEM images and corresponding vertical cross sections created by the focused ion beam. (**a**) Hematite Fe_2_O_3_ film before sulfidation, (**b**) marcasite α-FeS_2_ after sulfidation and (**c**) pyrite β-FeS_2_ after sulfidation. Deposited on FTO glass in DC mode, with the following parameters: discharge current 150 mA, pressure 4.7 Pa, distance from the nozzle 4 cm, argon flow rate 170 sccm, oxygen flow rate 1 sccm and deposition time 60 min (**a**) and 30 min (**b**,**c**).

The films were also analyzed by an SEM microscope equipped with a focused ion beam. A small platinum mask was deposited on top of the film. The ion beam then created the sharp cross-section below the mask edge (see Figure 7). The morphology of the film surface with pyramidal crystal shapes, as well as the columnar cross-section structure before (a) and after sulfidation (b), remained almost unchanged. In addition, the pyrite β-FeS_2_ in the black central part (c) is very similar to Figure 6, number 10, before sulfidation.

**Figure 8:**
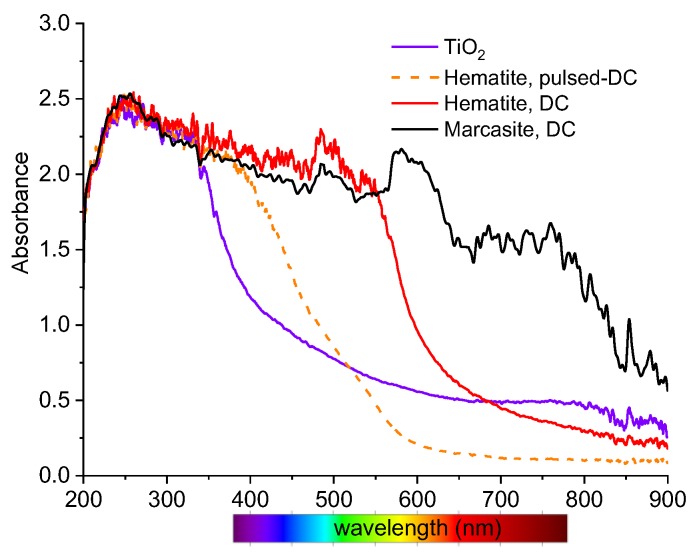
Absorbance spectra of hematite and marcasite films. TiO_2_ film shows for comparison.

Absorption spectra of the deposited films were measured in the range from 200 to 900 nm, using a deuterium lamp and an HR4000CG-UV-NIR spectrometer (originally Ocean Optics, now Ocean Insight, USA) and are shown in Figure 8. The absorbance of the reference TiO_2_ film starts to decrease at approximately 350 nm and up to 430 nm more than 90% of the radiation is absorbed. This edge is shifted up by approximately 50 nm for the hematite film deposited in the pulsed DC mode. A further shift up to 600 nm was observed for the red hematite region of the film deposited in the DC mode. This is significant improvement, since the solar radiation spectrum has a maximum at 500 to 600 nm. After sulfidation, the absorption of the marcasite film is extended to 800 nm. The magnetite and pyrite regions of the same films are almost nontransparent, and their absorbance is over the limits of the spectrometer used.

## 4. Conclusions

The ion energies of all positive ions, especially of Ar^+^ and Fe^+^, measured by the mass spectrometer with energetic analyzer showed energies below 3 eV. The addition of oxygen to the working gas inside the reaction chamber led to a shift of plasma potential to negative values. Raman spectroscopy clearly showed us a strong correlation between the color of the iron oxide film and its phase composition. A relatively clean pyrite phase was obtained from the magnetite, while the marcasite with admixture of the pyrite phase was obtained from the hematite. SEM images demonstrated low influence of sulfidation on the films’ microstructure. The sulfidation extended the light absorption to up to 800 nm.

## Figures and Tables

**Table 1:** Amplitudes of the strongest ion signal peaks in the measured mass spectra. Values are relative to that of the most abundant Ar^+^ ion, which is set to 100%. The ion energy was set to 0.5 eV. Distance from the cathode is 7 cm, Ar flow 150 sccm, O_2_ flow 3 sccm (last row only), discharge current 150 mA and pulsed mode duty cycle 10% at a frequency of 1 kHz.

Ion	H_3_^+^	O^+^	H_2_O^+^	Ar^++^	O_2_^+^	O_2_H^+^	ArH^+^	Fe^+^
Mass (amu)	3	16	18	20	32	33	41	56
Pulsed 5 Pa	—	—	—	15.5	—	—	2.2	1.4
Pulsed 20 Pa	9.6	—	—	11.2	—	—	21	12.2
DC 5 Pa	2.2	—	4.0	20.5	—	—	6.3	1.5
DC 5 Pa + O_2_	—	5.1	4.5	3.1	55	2.5	—	1.3

**Table 2:** Dominating phases identified in spectra of Figure 5 using the Raman spectra database RRUFF [16].

Before Sulfidation	After Sulfidation
(a) Magnetite Fe^2+^Fe^3+^_2_O_4_ (Fe_3_O_4_)	(d) Pyrite β-FeS_2_
(b), (c) Hematite Fe_2_O_3_	(e), (f) Marcasite α-FeS_2_

**Table 3:** EDX of samples with dominating phases. Line intensities were recalculated to weight and atomic percentages.

	Hematite		Magnetite		Marcasite		Pyrite	
Element	wt.%	at.%	wt.%	at.%	wt.%	at.%	wt.%	at.%
O (K line)	35.1	67.6	31.8	62.0	8.8	22.4	8.3	20.5
S (K line)	—	—	—	—	31.8	40.5	28.3	35.0
Sn (L line)	11.7	3.0	—	—	16.7	5.8	1.3	0.4
Fe (K line)	53.2	29.4	68.2	38.0	42.7	31.3	62.1	44.1
